# O066: Thermal disinfection of bedpans: European ISO 15883-3 guideline requirements are insufficient to ensure elimination of ARE and OXA-48 outbreak-strains

**DOI:** 10.1186/2047-2994-2-S1-O66

**Published:** 2013-06-20

**Authors:** LB van der Velden, MH Nabuurs-Franssen, A van Leeuwen, M Isken, A Voss

**Affiliations:** 1 Medical Microbiology and Infectious Diseases, Canisius Wilhelmina Hospital Nijmegen, The Netherlands; 2 Medical Microbiology, Radboud University Nijmegen Medical Center, Nijmegen, The Netherlands

## Introduction

During 2012, our hospital was faced with a vancomycin-resistant *Enterococcus faecium* (VRE) outbreak. Washer-disinfectors are used for the cleaning and thermal disinfection of bedpans. The European guideline NEN-ISO 15883-3 (ISO) states that washer- disinfectors have to achieve a minimum A_0_ value of 60 for appropriate disinfection of bedpans (≈ 80 °C for 60 seconds or 90 °C for 6 seconds). However, previous data have shown that some *E. faecium* strains survive 60 seconds at 80 °C. Following ISO, the A_0_ measurement should include a cold (a minimum interval of 60 minutes since the machine was last used) and hot start.

## Objectives

We determining the A_0_ value of the VRE outbreak-strain and the outbreak-strain of the OXA-48 *K.pneumoniae* outbreak in another Dutch hospital during 2011. Moreover the impact of a cold start measurement on the A_0_ value was evaluated.

## Methods

The minimum A_0_ value that results in the killing of all isolates was determined for both strains. Bacterial suspensions were heated at 65, 75 and 80 °C and samples for viable counts were obtained after 1,2,3 and 10 minutes at each temperature. VRE PCR and cultures were performed on bedpan swabs after disinfection; hot and cold start measurements were compared.

## Results

Adequate killing required a minimum A_0_ value of 180 for the VRE outbreak strain and 120 for the OXA-48 *K.pneumoniae*. The cold start resulted in a 30% lower A_0_ value, than the hot start. All washer-disinfectors in our hospital functioned in agreement with the European guideline, although the lowest A_0_ value was 73, only just above 60. Swabs taken from bedpans processed in these washers with low A_0_ values , were VRE-positive by PCR and cultures.

## Conclusion

Both outbreak strains survived the A_0_ value of 60 required in the ISO. VRE were identified by PCR as well as cultures of bedpans that had been disinfected by these washer-disinfector. We suggest to increase the minimal acceptable A_0_ value of washer-disinfectors to at least 180. Furthermore, the cold-start is needed for adequate A_0_-value measurement.

## Disclosure of interest

None declared.

